# The Excretion of Cisplatin after Hyperthermic Intrathoracic Chemotherapy

**DOI:** 10.3390/cancers15194872

**Published:** 2023-10-06

**Authors:** Christopher Larisch, Till Markowiak, Michael Ried, Dennis Nowak, Hans-Stefan Hofmann, Stefan Rakete

**Affiliations:** 1 Department of Thoracic Surgery, University Hospital Regensburg, 93053 Regensburg, Germany; 2 Institute and Clinic for Occupational, Social and Environmental Medicine, University Hospital, LMU Munich, 80539 Munich, Germany; 3 Comprehensive Pneumology Center Munich, German Center for Lung Research, 81377 Munich, Germany; 4 Department of Thoracic Surgery, Hospital Barmherzige Brueder, 93047 Regensburg, Germany

**Keywords:** hyperthermic intrathoracic chemotherapy, HITOC, excretion of cisplatin, malignant pleural mesothelioma, mesothelioma, thymoma, cisplatin, thoracic surgery, occupational exposure

## Abstract

**Simple Summary:**

Hyperthermic intrathoracic chemotherapy (HITOC) is an intraoperative treatment after a surgical cytoreduction of pleural malignancies. The pleural cavity is perfused with high doses of cytostatic drugs that are consequentially excreted via various body fluids. These are potential occupational health risk factors for medical staff and safety measurements must be established based on scientific evidence.

**Abstract:**

Hyperthermic intrathoracic chemotherapy (HITOC) is an additional intraoperative treatment option within the multimodality therapy of pleural malignancies. A chemotherapy perfusion with high-dose cisplatin is performed over a period of 60 min after surgical cytoreduction to improve local tumour control through the eradication of residual tumour cells. Although HITOC is increasingly used, there is only little scientific evidence about the necessary safety measures after HITOC. Therefore, the objective of this study was an analysis of cisplatin excretion via various body fluids after HITOC, with the aim of providing recommendations on occupational health and safety. Five patients undergoing HITOC were included. Before and after the HITOC, as well as during the following days, serum, urine, and bronchial secretion, as well as pleural effusion, were sampled. The platinum levels in the samples were measured using ICP-MS (inductively coupled plasma-mass spectrometry). Immediately after the HITOC, the mean levels of cisplatin increased dramatically in the serum (from 0.79 to 1349 µg/L), urine (from 3.48 to 10,528 µg/g creatinine), and bronchial secretion (from 0.11 to 156 µg/L). Thereafter, the cisplatin levels dropped to 133 µg/L in the serum and 994 µg/g creatinine in the urine within nine days after the HITOC. The AUC ratio shows 59% of the cisplatin being excreted via the urine after 48 h. The sampling of pleural effusion started 24 h after the HITOC, and the cisplatin levels decreased from 618 to 93 µg/L within nine days. Although the cisplatin levels in the body fluids of HITOC patients are much lower compared to patients receiving intravenous chemotherapy, a significant amount of cisplatin is excreted via these body fluids. Consequently, safety precautions must be implemented in the post-HITOC care of patients to avoid occupational exposure to cisplatin.

## 1. Introduction

Hyperthermic intrathoracic chemotherapy (HITOC) is an additional intracavitary treatment option within a multimodality therapy concept of malignant tumours of the pleura, mainly malignant pleural mesothelioma and thymic tumours with pleural spread. It is performed after surgical cytoreduction for better local tumour control. After surgical cytoreduction and closure of the chest, intracavitary heated (42 °C) chemotherapy perfusion with high-dose cisplatin is performed over a period of 60 min. The additional HITOC is intended to eradicate residual tumour cells in the pleural cavity after, at best, complete macroscopic tumour resection, and thereby to achieve improved local tumour control. Although HITOC should not be considered as experimental any longer, it has not been implemented in standard care yet. Nevertheless, there is increasing expertise and standardization. For example, there was a HITOC guideline set up in Germany in 2019. Despite there being many retrospective studies showing a benefit in survival due to HITOC, there is still no prospective trial investigating survival and comparing HITOC with other treatment regimens.

For intracavitary chemotherapy, cisplatin is used as standard in the highest possible dosage of up to 175 mg/m^2^ body surface area (BSA). The dosage of cisplatin seems to have an influence on the survival rate, as well as on the complication rate [1,2,3]. In particular, acute renal complications limit the cisplatin dosage [4]. Studies performed by our and other research groups confirmed extremely high cisplatin concentrations in perfusion fluid compared to low concentrations in patients’ serum [5,6,7,8,9]. Rusch et al. showed that the cisplatin in serum (and therefore urine) had a longer half-life than that in pleural fluid, suggesting a prolonged occupational risk for staff.

As a cytostatic drug, cisplatin is labelled as a presumable carcinogenic by the “International Agency for Research on Cancer” (IARC) [10]. Studies have revealed strong mutagenic [11,12,13], teratogenic [14,15], and carcinogenic [16,17] effects, mainly in living animal models. There are short-term and long-term clinical effects of these: in addition to an increased risk for secondary malignancies, there are increased risks for cardiovascular complications, pulmonary toxicity, nephrotoxicity, ototoxicity, hypogonadism, and infertility [18] etc. Apart from a rather short interval of exposure with a high dose in the course of chemotherapy, the effect of longstanding (meaning over years) low-level exposure to cytostatic drugs in humans remains not fully clear and very difficult to investigate.

During and after HITOC, there are several possibilities for staff to come into contact with the cisplatin excreted by the patient: via urine (while emptying the urine catheter bag), bronchial secretion (while coughing, performing oral hygiene, or endotracheal suction), pleural fluid in the thoracic drainages, and pleura fluid running aside the drainage out of the wound. Thus, unintended contact with cisplatin and other cytostatic drugs by the surgical or nursing staff should be avoided, and special safety actions should be applied, such as the use of cytostatic hand gloves, fluid-repellent surgical gowns, safety glasses with side protection, and the signposting of the operation theatre and the immediate surroundings of the patient’s bed [19]. However, cisplatin exposure during and after HITOC has not yet been fully investigated, especially with regard to occupational safety.

Therefore, the objective of this study was to measure the cisplatin concentrations in different body fluids (serum, urine, pleural fluids, and bronchial secretion) after HITOC with the aim of providing a better understanding of the occupational health and safety risks concerning HITOC. This could enable the recommendation of more evidence-based measurements in the future.

## 2. Materials and Methods

### 2.1. Study Design

This was a prospective study in collaboration with the departments of Thoracic Surgery of the University Hospital Regensburg and the Hospital Barmherzige Brüder in Regensburg and the Institute and Clinic for Occupational, Social and Environmental Medicine at the University Hospital of the LMU Munich. The study was approved by the local Ethical Committee of the University of Regensburg (reference number: 21-2419-101) and was conducted in accordance with the Declaration of Helsinki (as revised in 2013). The time frame was February 2021 until August 2022. All the patients signed informed consent before participating in the study. The inclusion criteria were patients with cisplatin-based HITOC after a radical surgical cytoreduction with normal preoperative serum creatinine levels or no diagnosis of renal insufficiency, respectively.

The primary endpoint of this study was the measurement of cisplatin in the serum, as well as the cisplatin excreted by the patient through bronchial secretion, urine, and pleural fluid over time. Secondary recommendations regarding occupational health and safety should be given based on our data.

A comparative statistical analysis was not the goal of this study, but rather a first-time investigation of the questions. Five patients were included. The small number of participants is explained by the infrequent use of HITOC despite us being a renowned centre and is due to the study design: this was an exploratory study only.

### 2.2. Surgery and Hyperthermic Intrathoracic Chemotherapy (HITOC)

As primary pleural malignancies, e.g., malignant pleural mesothelioma, are usually unilateral diseases, surgical therapy, including HITOC, is performed unilaterally only. Macroscopic cytoreduction was performed by a radical pleurectomy/decortication of the tumour and the pleura, partly extended by a resection of the pericardium and diaphragm or a lung-sparing resection (eP/D). In one case, extrapleural pneumonectomy (EPP) was necessary. The HITOC was performed according to the inhouse standard and was not altered by the study design. Preoperatively, all patients received a transurethral catheter, which was kept for several days after surgery. The perfusion with cisplatin was performed via one inflow and three outflow tubes. After the connection of the tubes with the perfusion system, the thorax was filled with approximately four litters of physiologic solution. After establishing stable circulation and a perfusion temperature of approximately 42 °C, cisplatin with a dose of 175 mg/m^2^ BSA was added, and the thoracic cavity was perfused over 60 min. During the HITOC, the staff wore special cytostatic hand gloves, a pair of glasses with side protection, and a fluid-repellent surgical gown.

### 2.3. Sample Collection

The operation and the HITOC with the sampling collection took place in the Department of Thoracic Surgery of the University Medical Center Regensburg. The measurements of platinum and the analysis of the raw data were performed at the Institute and Clinic for Occupational, Social and Environmental Medicine of the LMU University Munich.

**Figure 1:**
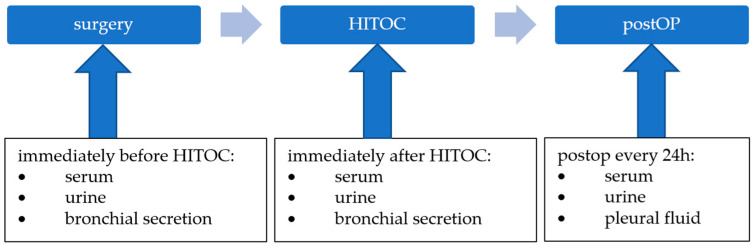
Overview of timing of sample collection.

Immediately before starting the HITOC, 50 mL of urine (via the urinary catheter), 5 to 10 mL of bronchial secretion (via an endobronchial suction catheter), and a serum sample were taken. This was repeated immediately after finishing the HITOC and subsequently every 24 h for nine days postoperatively, particularly for the urine, serum, and pleural fluid (Figure 1). The serum samples were gathered in regular serum monovettes during regular blood work. The urine and pleural fluid samples were gathered by taking fresh urine and pleural fluid, respectively, directly from the tubes.

### 2.4. Reagents and Chemicals

All the reagents were of the highest available grade. Concentrated hydrochloric acid for an ultra trace elemental analysis was obtained from Fisher Scientific (OptimaTM, Waltham, MA, USA). Ultrapure water (resistivity: 18.2 Ω) was provided by a water purification system (Direct-Q^®^, Merck, Darmstadt, Germany). The platinum standard (1000 mg/L) was obtained from Merck (Darmstadt, Germany). Lyophilized control material for the serum (lot 1399, level 1) and urine (lot 1227, level 1 and 2) were obtained from Recipe (ClinChek^®^, Munich, Germany) and prepared according to the manufacturer’s instructions.

### 2.5. Instrumentation

All the samples were analysed on an inductively coupled plasma tandem mass spectrometer, ICP-MS (8900, Agilent Technologies, Waldbronn, Germany), equipped with a SPS-4 autosampler connected to an Ultra High Matrix Introduction (UHMI) interface. The instrument was tuned daily to achieve the optimum sensitivity, oxide ratio, and doubly charged ratio. All the samples were analysed in HMI mode using a dilution factor of four. All the analyses were run in single quadrupole mode, and helium was used as a collision gas to reduce polyatomic interferences. 195Pt was quantified using external calibration and 159Tb was used as an internal standard.

### 2.6. Urine Analysis

**Figure A1:**
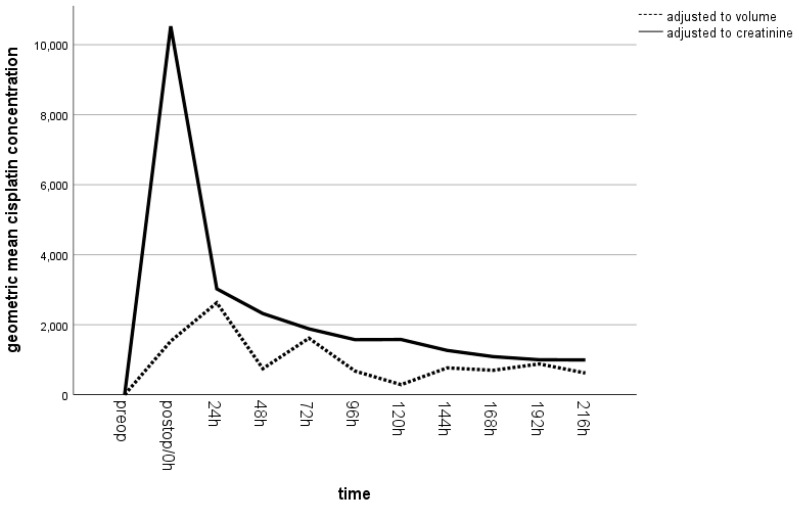
Mean cisplatin concentration in urine dependent of time. Comparison of values adjusted to urinary creatinine (red; µg cisplatin/g creatinine) or urinary volume (blue; µg cisplatin/L urine). There is not such a smooth decline in cisplatin levels in the volume-adjusted group.

**Figure A2:**
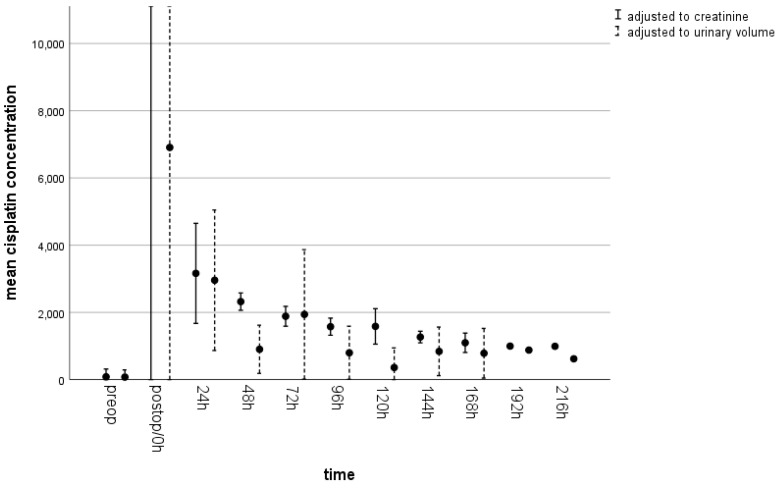
Error bars of mean cisplatin concentration in urine (red: µg cisplatin/g creatinine; blue: µg cisplatin/L urine). There is a very strong spread immediately after HITOC (postop/0 h). In general, the spreads in the creatinine-adjusted group tends to be smaller. Thus, these values seem to be more reliable.

The measurements of the substances in the urine need a reference. This could be the volume of the urine (for example millilitres or litres) or a physiologic reference substance (mostly urinary creatinine). Both units have their weaknesses, as they depend on the current renal function and volume status along with other factors. For example, creatinine-adjusted cisplatin levels could be exaggerated by a low creatinine clearance, such as during acute renal impairment. On the other hand, volume-adjusted cisplatin levels are highly dependent on volume status and fluid administration. We decided to show both units for a better comparison with other studies. In the results, we only go into detail concerning creatinine-adjusted cisplatin levels, as we found a lesser variability in this group. Please see the appendix (Appendix A, Figure A1 and Figure A2) for further visualization.

The creatinine in the urine samples was analysed by the Institute of Laboratory Medicine at the University Hospital of the LMU Munich on a Cobas C702 instrument using the Jaffé method.

### 2.7. Sample Preparation and Analysis

The samples were thawed on a roll mixer until they reached room temperature. A sample aliquot of 200 µL was diluted with 1800 µL of 0.3% (*v*/*v*) hydrochloric acid containing 10 µg/L of internal standard. The standards and control material were diluted in the same manner. The limit of quantitation (LOQ) in the urine was 0.001 µg/L. All the analyses were carried out in duplicate. One bronchial secretion sample could not be pipetted after thawing due to its high viscosity. Furthermore, not every sample was available for all the patients at all times.

### 2.8. The Rational of the Measurement of Platinum and Cisplatin 

The platinum levels were analysed using ICP-MS (inductively Coupled Plasma—Mass Spectrometry) and converted into cisplatin levels by using a multiplication factor of 1.54 (using the molar equation). This is possible under the condition that there is no preexisting platinum in the body. Recent research has suggested that intravenously applied cisplatin is present as cisplatin and its monohydrated complex in the blood [20].

### 2.9. Statistical Analysis

First, the data were collected in tabular form using Microsoft Excel, Version 15.0, and Office 365. The urinary cisplatin levels were adjusted to creatinine for intra- and interindividual comparisons. Descriptive statistics and visualization were performed using IBM SPSS Statistics 29. Due to the small sample size with non-normally distributed data and the large spread of platinum levels in the samples, non-parametric testing was applied using the geometric mean, minimum, and maximum for descriptive data.

## 3. Results

### 3.1. General Patients’ Data and Overview

Please see Table 1 for general clinical data of the patients included.

In Table 2, you can view the separate geometric mean cisplatin concentrations for a better overview.

### 3.2. Cisplatin in Serum

**Figure 2:**
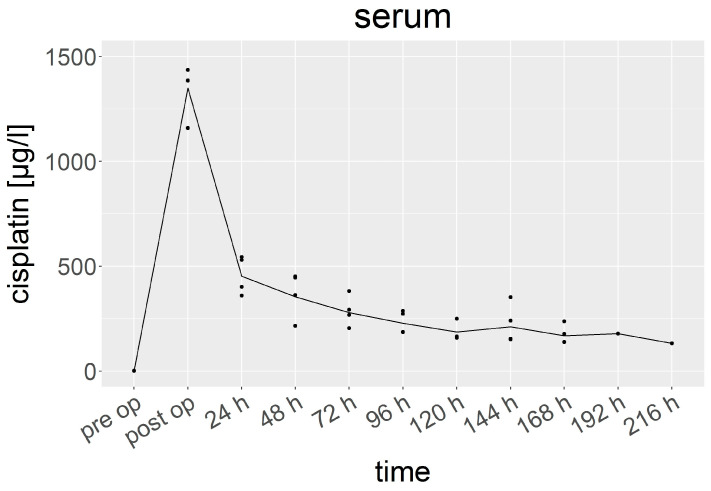
Cisplatin concentration in serum (µg/L). Specified hours refer to the time of sampling after HITOC. The dots represent individual results.

The cisplatin levels in the serum ranged from 0.3 to 1437 µg/L (Figure 2). Immediately after the HITOC, the mean levels of cisplatin dramatically increased from 0.8 (0.33–1.90) µg/L to 1349 (1160–1437) µg/L. Within 24 h after the surgery, the levels dropped to approximately one third of the peak concentration with 452 (360–544) µg/L. Thereafter, a first-order kinetics elimination of cisplatin was observed until nine days after the HITOC (t1/2 ≈ 90 h). The measured cisplatin amounts were 354 (216–452) µg/L at 48 h and 279 (204–381) µg/L at 72 h, finally reaching 133 µg/L at 216 h in the end.

It is worth mentioning that the cisplatin levels in the serum and pleural fluid (where data gathering only started 24 h postoperatively) were very similar.

### 3.3. Cisplatin in Pleural Fluid

**Figure 3:**
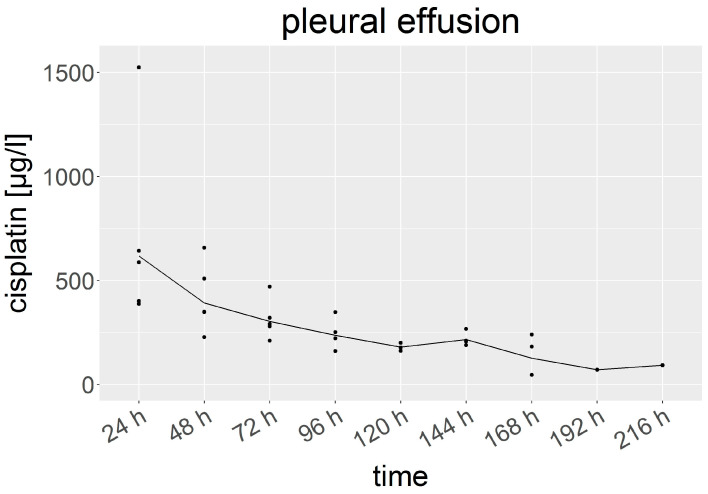
Cisplatin concentration in the pleural effusion (µg/L). Specified hours refer to the time of sampling after HITOC. The dots represent individual results.

After the HITOC, the pleural fluid was sampled every 24 h, beginning at 24 h after surgery. Here, the cisplatin levels ranged from 47 to 1524 µg/L (Figure 3). There was a gradual decline from the mean of 618 (388–1524) µg/L after 24 h to 392 (228–658) µg/L at 48 h and 304 (211–471) µg/L at 72 h, finally reaching 93 µg/L in the end. Similar to in the serum and urine, the cisplatin elimination followed a first-order kinetics (t1/2 ≈ 52 h).

### 3.4. Cisplatin in Urine

**Figure 4:**
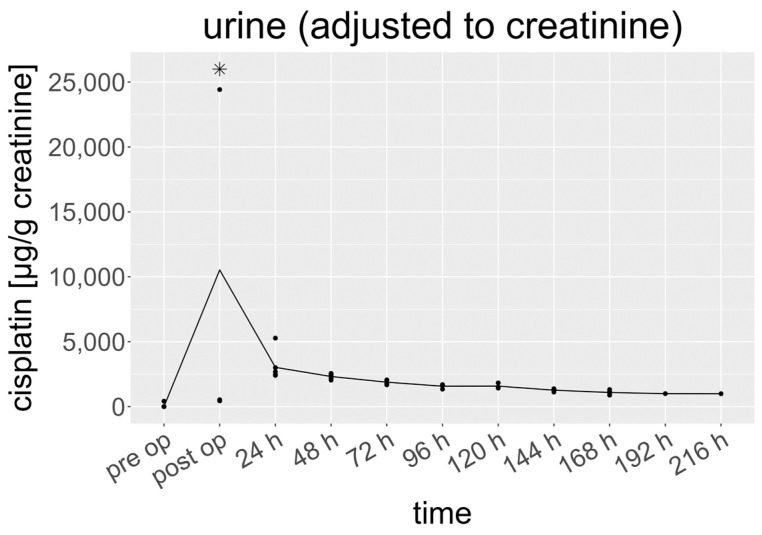
Cisplatin concentration in urine, adjusted to creatinine (µg/g creatinine). Specified hours refer to the time of sampling after HITOC. The dots represent individual results. For clarity, two values (114,233 and 197,421 µg/g creatinine) at “postop”) are not shown separately. * represents a statistical outlier.

The creatinine-adjusted cisplatin levels in the urine ranged from 0.15 to 197,421 µg/g creatinine (Figure 4). Similar to the serum, the mean levels of cisplatin strongly increased from 3.5 (0.15–417) to 10,528 (442–197,421) µg/g creatinine after the HITOC. In contrast to the other sampling times, the cisplatin levels in the samples taken immediately after the HITOC showed a much higher variation and ranged from 442 to 197,421 µg/g creatinine. The mean cisplatin levels in the urine decreased by 71% to 3025 (2404–5278) µg/g creatinine within 24 h after surgery. After 24 h, the concentrations declined at a slower rate afterwards (t1/2 ≈ 83 h) to 2319 (2040–2554) µg/g creatinine at 48 h and 1882 (1677–2059) µg/g creatinine at 72 h, finally reaching 994 µg/g creatinine in the end.

Considering the AUC ratio ([AUC(0;48 h)/AUC(0;216 h)]), a relative portion of 59% of creatinine-adjusted cisplatin was excreted after 48 h. After 96 h, 75% was excreted.

### 3.5. Cisplatin in Bronchial Secretion

**Figure 5:**
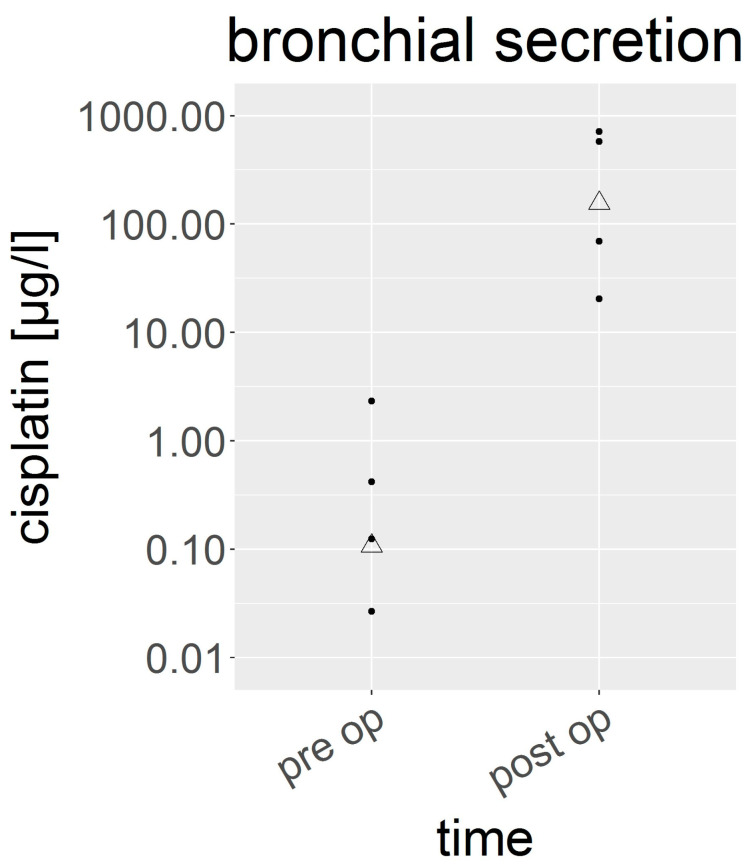
Cisplatin concentration in the bronchial secretion (µg/L) on a logarithmic scale, in dependence of time. The dots represent individual results. The triangles represent the geometric mean.

Samples of bronchial secretion were available immediately before and after the HITOC, as all the patients were extubated at the end of surgery. The cisplatin levels ranged from 0.004 to 713 µg/L (Figure 5). Again, the mean levels significantly increased after surgery: from 0.11 (0.004–2.33) µg/L to 156 (70–580) µg/L.

## 4. Discussion

This was the first study evaluating the postoperative cisplatin levels in body fluids after HITOC. Recent studies have mainly concentrated on intraoperative and postoperative data about HIPEC (hyperthermic intraperitoneal chemotherapy), which is more broadly used than HITOC. It is clear that contamination with carcinogenic substances should be as low as possible to avoid circumstantial exposure. The most likely route of occupational exposure to cytostatic drugs in the health are setting is transdermal uptake [21,22]. Consequently, basic safety measures include special gloves, special gowns, and glasses.

Performing HITOC, staff face two different challenges. On the first hand, it is a surgical task to create tight wound sutures and a sealed perfusion system in order to not let cisplatin be spilled during the HITOC, as the leakage of cytostatic drugs has been described in the literature [10]. After the HITOC, there is the second problem of cisplatin inevitably being excreted by the patient through the urine, bronchial secretion, and thoracic drainages.

In a previous study conducted by our working group, we measured the amount of cisplatin on the surface of the surgeon’s and the perfusionist’s hand gloves, as well as on the display of the perfusion system, on the floor in the operating theatre, and the inside of the endobronchial tube. The highest burden of cisplatin—although very low generally speaking—was detected on the surgeon’s hand gloves (median 1.73 pg cisplatin/cm^2^), followed by the perfusionist’s hand gloves (0.69 pg cisplatin/cm^2^) and the display of the perfusion system (0.57 pg cisplatin/cm^2^) [19]. In a comparable study focusing on HIPEC (hyperthermic intraperitoneal chemotherapy), the median cisplatin contamination on surfaces was 2.31 pg cisplatin/cm^2^ (the platinum level multiplied by 1.54 for the cisplatin level), and thereby three times higher than that on the highest contaminated area in our study. The cisplatin contamination on the surgeon’s gloves was from 0.03 to 729 ng per sample (which makes a comparison difficult) [10]. Another study evaluated cisplatin contamination by air and on the floors in the operating room after open HIPEC. While uptake by air was not detectable, there were low amounts of cisplatin on the floor [23]. A study concentrating on PIPAC (pressurized intraperitoneal aerosol chemotherapy) found a low air contamination with platinum, and the median cisplatin contamination on the surgeon’s gloves was 0.89 pg cisplatin/cm^2^ [24]. Air contamination is likely explainable, as cisplatin is aerosolized during this particular procedure. Another study investigated the platinum contamination in the urine of healthcare workers being engaged intraoperatively during a HITOC procedure. There was no platinum found in the urine, despite heavy contamination of the operating table, floor, and overshoes [25]. Similarly, one study showed urinary platinum to be below the limit of measurability in over 50% of workers engaged in HITOC, and there was no significant difference in the platinum levels in comparison to a control group [26]. In contrast, one study measured 33% of blood samples as positive for platinum compounds in one surgeon performing HIPEC over the course of three years [27].

The main place where medical staff are in daily contact with cytostatic drugs is the oncological ward. Several studies have looked for platinum contamination here. In one study, no traces of platinum compounds were found in almost 400 healthcare workers on an oncological ward, despite the contamination of several surfaces on the ward itself [28]. Another study demonstrated that, in urinary samples, the platinum levels were below measurability [29].

Now, we took the next step to answering the second issue: the cisplatin levels showed a steep decline in the serum and urine 24 h postoperatively, followed by a gradual decrease over the next days. Oppositely, the cisplatin levels in the pleural fluid decreased linearly. The serum levels were very low, as already shown in a previous study by our working group [5]. The fact that the cisplatin levels in the serum and pleural fluid were similar is of special interest, as it could be used for future research.

Although the cisplatin levels in the urine were rather high—as cisplatin is renally excreted—they were also low compared to intravenous application during conventional chemotherapy [29]. This would be an explanation for the infrequent incidence of renal failure, despite the intrathoracic application of very high cisplatin doses. Additionally, in our department, we implemented a nephroprotection treatment using fluid balancing and sodium thiosulfate. 

A very comparable study evaluated the platinum contamination on an intensive care unit after HIPEC. As the researchers measured platinum, we converted these results into cisplatin using a molar equation (see also Section 2.8 please) for an easier comparison. The median cisplatin concentration in the urine was 1940 pg/mL (which is equivalent to µg/L) on the first postoperative day and 636 pg/mL on the third day, compared to our higher doses of 2627 and 1617 µg/L, respectively. In the drainage fluids, the cisplatin levels were generally lower, reaching from 1047 pg/mL on the first day to 490 pg/mL on the third day, compared to our data of 618 and 304 µg/L, respectively. One can conclude that, in both cases, there were steep declines in the cisplatin levels. However, exact comparisons are difficult, as they used medians and we used geometric means. Furthermore, the cisplatin dose in HIPEC is generally lower than that in HITOC. In this particular paper, the authors applied a cisplatin dose of approximately from 65 to 90 mg/m^2^ BSA (body surface area), whereas we used a dose of 175 g/m^2^ BSA, meaning twice the dose. The median platinum contamination found on the nurses’ gloves was 0.2 pg/cm2, and thus lower than that for staff exposed intraoperatively [30].

It is to be explained why there was a cisplatin contamination at baseline in our data although no patient received neoadjuvant chemotherapy. One explanation is possible contamination in the course of the laboratory process. Another explanation would be that there is a platinum burden on every human being (due to modern industrial life, e.g., platinum is used in catalysts), which could explain the preoperative “cisplatin” levels (as we converted the platinum in the cisplatin data using the molar equation). Summarizing our results and the current literature, we recommend applying safety measures for at least the first 48 h after HITOC, followed by an exchange of the chest tube boxes and the bag of the urinary catheter on the third day. This is supported by the result that, according to the AUC ratio, roughly 60% of cisplatin is excreted via the urine by that time.

This study comprises some limitations. First, it was a study with only a small sample size, which is prone to outliers. However, the study was of an exploratory and not a conforming manner. Second, we measured platinum as a surrogate parameter for cisplatin. As this should be scientifically correct concerning endobronchial and pleural fluids, as well as early serum and urine samples, the late serum and urine samples could contain more metabolites of cisplatin, which may be less toxic and would thus constitute a lower risk for the staff. Third, measuring urinary concentrations comprises weaknesses, with the possibility of either overstating or understating the real excretion. Fourth, we did not measure the platinum levels in the patients’ sputum following the sampling of the endobronchial secretion. Therefore, we cannot give a recommendation, e.g., concerning safety measures for nursing staff performing dental hygiene on the patient.

Additionally, future research could measure the cisplatin contamination of patients’ skin (especially in the thoracic area and at the exit locations of the chest tubes), as nursing staff care particularly about these body parts of patients after thoracic surgery. Furthermore, samples should be taken from additional locations of the patient’s surroundings in the ICU, e.g., perfusor pumps and patients’ desks, as there could be considerable amounts of cisplatin due to cross contamination.

## 5. Conclusions

Taken together, the cisplatin levels in the serum and urine after HITOC were much lower compared to those of patients who received conventional intravenous chemotherapy. Nevertheless, safety precautions must be implemented in the post-HITOC care of patients to avoid occupational exposure to cisplatin. We suggest applying safety measures in the first postoperative 48 h, followed by an exchange of the chest tube box and the urinary catheter bag to remove a great part of the excreted cisplatin from the direct working area.

## Figures and Tables

**Table 1:** General patients’ data (MPM: malignant pleural mesothelioma; EPP: extrapleural pneumonectomy; and eP/D: extended pleurectomy/decortication).

Patient	Age (Years)	Sex	Tumour Stage	Surgery	Creatinine (mg/dL)/GFR Preop
1	39	male	MPM stage II	EPP	0.64/125
2	57	male	MPM stage II	eP/D	0.65/110
3	75	female	MPM stage IB	eP/D	0.76/77
4	74	male	MPM IIIA	eP/D	0.85/86
5	64	male	MPM stage IB	eP/D	0.75/98

No patient received neoadjuvant chemotherapy.

**Table 2:** Geometric mean cisplatin concentrations (minimum/maximum) in serum, pleural effusion, urine, and bronchial secretion. * means that there was only one valid measurement. “---” means that there was no (valid) measurement.

Time	Serum (µg/L)	Pleural Effusion (µg/L)	Urine (µg/g Creatinine)	Urine (µg/L Urine)	Bronchial Secretion (µg/L)
preop	0.79 (0.33–1.90)	---	3.5 (0.15–417)	1.29 (0.07–384)	0.11 (0.004–2.33)
postop/0 h	1349 (1160–1437)	---	10,528 (442–197,421)	1531 (143–27,873)	156 (70–580)
24 h	452 (360–544)	618 (388–1524)	3025 (2404–5278)	2627 (1490–5637)	---
48 h	354 (216–452)	392 (228–658)	2319 (2040–2554)	745 (305–1630)	---
72 h	279 (204–381)	304 (211–471)	1882 (1677–2059)	1615 (516–3159)	---
96 h	229 (186–287)	237 (161–348)	1573 (1344–1696)	676 (371–1329)	---
120 h	187 (159–250)	180 (162–201)	1578 (1424–1828)	288 (102–561)	---
144 h	211 (152–353)	216 (189–268)	1266 (1122–1377)	767 (513–1509)	---
168 h	168 (138–237)	127 (47–241)	1088 (882–1314)	698 (401–1456)	---
192 h	178 *	71 *	999 *	879 *	---
216 h	133 *	93 *	994 *	620 *	---

## Data Availability

Data are available on request due to privacy and ethical restrictions.
